# Predictive ability of obesity- and lipid-related indicators for metabolic syndrome in relatively healthy Chinese adults

**DOI:** 10.3389/fendo.2022.1016581

**Published:** 2022-11-18

**Authors:** Yuting Duan, Weiguang Zhang, Zhe Li, Yue Niu, Yizhi Chen, Xiaomin Liu, Zheyi Dong, Ying Zheng, Xizhao Chen, Zhe Feng, Yong Wang, Delong Zhao, Qiu Liu, Hangtian Li, Huifang Peng, Xuefeng Sun, Guangyan Cai, Hongwei Jiang, Xiangmei Chen

**Affiliations:** ^1^ Henan Key Laboratory of Rare Diseases, Endocrinology and Metabolism Center, The First Affiliated Hospital, and College of Clinical Medicine of Henan University of Science and Technology, Luoyang, China; ^2^ National Clinical Research Center for Kidney Diseases, State Key Laboratory of Kidney Diseases, Beijing Key Laboratory of Kidney Disease Research, First Medical Center of Chinese PLA General Hospital, Nephrology Institute of the Chinese People’s Liberation Army, Beijing, China; ^3^ Department of Nephrology, Hainan Hospital of Chinese PLA General Hospital, Hainan Province Academician Team Innovation Center, Sanya, China

**Keywords:** metabolic syndrome, lipid accumulation product, obesity, lipid, adults, healthy

## Abstract

**Background and objective:**

Metabolic syndrome (MetS) is an important risk factor for cardiovascular complications and kidney damage. Obesity- and lipid-related indices are closely related to MetS, and different indices have different predictive abilities for MetS. This study aimed to evaluate the predictive value of eight obesity- and lipid-related indicators, namely, body mass index (BMI), lipid accumulation product (LAP), body roundness index (BRI), Chinese visceral adiposity index (CVAI), body adiposity index (BAI), abdominal volume index (AVI), triglyceride glucose index (TYG), and visceral adiposity index (VAI), for MetS.

**Methods:**

A total of 1,452 relatively healthy people in Beijing were enrolled in 2016, and the correlation between the eight indicators and MetS was analyzed by multivariate logistic regression. The receiver operating characteristic (ROC) curve and the area under the curve (AUC) were used to analyze the predictive ability of the eight indicators for MetS. The Delong test was used to compare the AUC values of the eight indicators. MetS was defined according to the Chinese Guidelines for the Prevention and Treatment of Type 2 Diabetes (2020 edition), the revised National Cholesterol Education Program Adult Treatment Group (NCEP-ATPIII), and the International Diabetes Federation (IDF).

**Results:**

Using these three sets of criteria, LAP, TYG, CVAI, and VAI, which are based on blood lipids, had higher AUC values for MetS prediction than BMI, BRI, AVI, and BAI, which are based on anthropometry. LAP had the highest AUC values of 0.893 (0.874–0.912), 0.886 (0.869–0.903), and 0.882 (0.864–0.899), separately, based on the three sets of criteria.

**Conclusion:**

The eight obesity- and lipid-related indicators had screening value for MetS in relatively healthy people, and of the eight indicators, LAP performed the best.

## Introduction

Metabolic syndrome (MetS) is a group of conditions characterized by cardiometabolic risk, including obesity (especially central obesity), elevated blood pressure, elevated blood glucose, elevated triglyceride (TG), and decreased high-density lipoprotein cholesterol (HDL-c) levels (1). These are important risk factors for atherosclerotic cardiovascular disease (CVD) and type 2 diabetes (T2DM), which can lead to severe complications, such as arteriosclerosis, decreased renal function, myocardial infarction, and cerebral infarction (2–4). Therefore, screening for MetS in relatively healthy people is of great significance in understanding their disease status or predisease status in advance, preventing related diseases caused by MetS in advance, and reducing the waste of public health resources and the medical burden.

Visceral fat accumulation is an important feature of MetS. However, the gold standards for assessing visceral fat, such as magnetic resonance imaging (MRI) and computed tomography (CT), involve exposure to radiation or are expensive and time-consuming. People are starting to use simple measures to assess visceral fat. Body mass index (BMI) is the most common anthropometric index used in epidemiological and clinical studies to classify overweight and obesity but is affected by differences in age, sex, and race and does not distinguish between fat and muscle mass (5, 6). Therefore, a variety of obesity- and lipid-related indicators have gradually been developed to assess visceral fat and predict MetS.

The body roundness index (BRI), body adiposity index (BAI), and abdominal volume index (AVI) are all new anthropometric indicators that can be used to effectively evaluate visceral obesity and make up for the deficiencies of BMI (7–9). The BRI has shown a superior ability to predict atherosclerosis in overweight/obese people (10), and it also does well in predicting MetS (11). The BAI has been shown to predict hypertensive events and screen for coronary heart disease risk (12, 13). The AVI reflects visceral fat content by assessing total abdominal volume, which is associated with impaired glucose tolerance (IGT) and diabetes mellitus (DM) (9, 14) and has strong predictive power for MetS in adolescents (15). The lipid accumulation product (LAP), visceral adiposity index (VAI), Chinese visceral adiposity index (CVAI), and triglyceride glucose index (TYG) are recently developed indices for estimating visceral fat based on a combination of abdominal obesity index [waist circumference (WC), BMI], blood glucose, and circulating lipids (HDL-C, TG) (6, 16–18). The LAP and TYG play an important role in identifying DM and prediabetes mellitus (19) and have a good ability to predict MetS (20–22). Both the VAI and CVAI can be used as markers of cardiometabolic risk (16, 23). All of these indicators show certain predictive power for MetS, but the best indicator to evaluate MetS is still controversial. The purpose of our study was to evaluate the performance of eight obesity- and lipid-related indicators (BMI, LAP, BRI, CVAI, BAI, AVI, TYG, and VAI) in predicting MetS in a relatively healthy population in China under three sets of criteria. Meanwhile, we were in search of the best sole indicator among the eight indicators to predict MetS.

## Materials and methods

### Study design and participants

The study was conducted at the Chinese PLA General Hospital in 2016 and recruited volunteers from Beijing, China. In this study, 2,217 volunteers aged ≥18 years were initially recruited. A total of 765 subjects were excluded according to the following exclusion criteria (**Supplementary Figure 1**): a) those with respiratory diseases, such as chronic obstructive pulmonary disease, asthma, bronchiectasis, etc.; b) those with musculoskeletal disease or rheumatologic disease, such as sarcopenia, fracture, rheumatoid arthritis, etc.; c) those with one of the following diseases in the previous 6-month period: liver cirrhosis, stroke, myocardial infarction, and malignant tumor; d) those unable to cooperate with the tests and sample collection; and e) those lacking the required data. Ultimately, 1,452 people were included in the study. This study was performed in accordance with the Declaration of Helsinki and approved by the Ethics Committee of the Chinese People’s Liberation Army General Hospital. All the participants provided signed informed consent and agreed to participate in this survey.

The information collected in this study included sociodemographic characteristics, medical history, family history, laboratory tests, etc. Anthropometric data, including weight, height, WC, hip circumference, and blood pressure, were measured by professional researchers according to standard protocols. The participants wore light clothing and were barefoot when their weight and height were measured. WC was measured using a flexible plastic tape measure at the navel level after the patient exhaled, and hip circumference was measured at the widest part of the hip. Blood pressure was measured in the participant’s non-dominant arm using automated electronic equipment; after a 5-min rest, blood pressure was measured in a 1-min interval thrice. The mean systolic and diastolic blood pressures of the three readings were recorded using a questionnaire.

### Biochemical measurements

Participants fasted for at least 8 h for the collection of venous blood to measure fasting blood glucose (FBG), creatinine (Cr), total cholesterol (TC), TG, HDL-C, low-density lipoprotein cholesterol (LDL-C), and other biochemical indicators. The estimated glomerular filtration rate (eGFR) was calculated using the Chronic Kidney Disease Epidemiology Collaboration (CKD-EPI) equation. The formulas for calculating BMI, LAP (24), BRI (7), CVAI (17), BAI (8), AVI (9), TYG (18), and VAI (16) are shown in **Supplementary Table 1**.

### Definition of MetS

MetS was defined according to the Chinese Guidelines for the Prevention and Treatment of Type 2 Diabetes (2020 edition) (25), the revised National Cholesterol Education Program Adult Treatment Group (NCEP-ATPIII) (26), and the International Diabetes Federation (IDF) (27) (**Supplementary Table 2**).

### Statistical analysis

The normal distribution of variables was assessed by the Kolmogorov–Smirnov test. The homogeneity of variance was assessed by the Levene test or one-way ANOVA. Categorical variables are presented as percentages, and continuous variables are described as the mean ± standard deviation (SD) for normally distributed data or the median (interquartile range) for skewed data. Comparisons between groups were performed using the Student’s *t*-test, the chi-square test, or the Mann‐Whitney *U* test. Binary logistic regression analysis was used to assess the relationship between obesity- and lipid-related indices and the incidence of MetS. Data were summarized as odds ratios (ORs) and regression coefficients [95% confidence intervals (CIs)]. The ORs indicated the change in the odds per unit increase in the anthropometric measures. When performing binary logistic regression, adjustments were made for the participants’ age, systolic blood pressure, diastolic blood pressure, TC, and eGFR. Adjusted variables were diagnosed by collinearity according to the following criteria: variance inflation factor (VIF) >10 or tolerance of approximately 0.1, condition index >30, and variance ratio >50%. Selected variables were not collinear. Receiver operating characteristic (ROC) analysis was used to compare the diagnostic performance of logistic models. Internal ten-fold cross-validation and penalty regression for validation. The tuning of the hyperparameters lambda and alpha was done through grid search, and the best models were reported in different groups with the highest mean validation AUC. The source codes were posted on github (https://github.com/yotasama/cv.elasticnet.r).

The ROC of the sole index analysis was used to compare the diagnostic performance of obesity- and lipid-related indices for MetS. Youden’s index (sensitivity + specificity − 1) was used to determine the optimal cutoff point of each indicator. All statistical analyses were performed using R.4.2.0 with package glmnet v4.1-4 and IBM SPSS statistical software, version 25 (IBM Corporation, Armonk, New York, NY, USA). The AUC values of all indicators were compared using the DeLong test and calculated using MedCalc Version 19.0 software (Ostend, Belgium). Differences were considered statistically significant at *P*-values of <0.05.

## Results

### General characteristics of the participants

The demographic characteristics, anthropometric measurements, and obesity- and lipid-related indices are presented in **Table 1**.** A** total of 1,425 subjects were enrolled, consisting of 615 men with an average age of 58.07 ± 13.57 years and 837 women with an average age of 58.26 ± 13.25 years.

**Table 1:** Basic characteristics of the participants.

Variable	Male	Female	Total
*N*	615	837	1,452
Age (years)	58.07 ± 13.57	58.26 ± 13.25	58.18 ± 13.38
Height (cm)	171.26 ± 5.61	159.48 ± 5.50	164.47 ± 8.04
Weight (kg)	73 ± 12.04	61.65 ± 9.85	66.46 ± 12.20
WC (cm)	91.3 ± 9.11	83.39 ± 9.35	86.74 ± 10.04
HC (cm)	100.0 ± 5.85	97.99 ± 6.79	98.83 ± 6.48
BMI (kg/m^2^)	24.84 ± 3.6	24.26 ± 3.91	24.50 ± 3.79
TG (mmol/L)	1.38 (0.93, 1.97)	1.23 (0.93, 1.72)	1.29 (0.93, 1.83)
TC (mmol/L)	4.63 ± 0.93	4.84 ± 0.94	4.75 ± 0.94
HDL-C (mmol/L)	1.31 ± 0.34	1.53 ± 0.38	1.44 ± 0.38
FPG	5.57 ± 1.70	5.43 ± 1.39	5.49 ± 1.53
SBP (mmHg)	126.6 ± 14.7	124.16 ± 17.32	125.17 ± 16.30
DBP (mmHg)	75.44 ± 9.63	70.91 ± 10.06	72.83 ± 10.13
eGFR (ml/min/1.73 m^2^)	91.61 ± 19.0	89.13 ± 17.19	90.18 ± 18.00
LAP	35.4 (20.79, 58.95)	32.67 (18.94, 48.32)	34.05 (19.75, 52.78)
BRI	4.63 ± 1.04	3.88 ± 1.24	3.96 ± 1.16
CVAI	120.16 (93.19, 146.66)	101 (70.69, 124.72)	110.26 (78.93, 133.95)
AVI	16.91 ± 3.27	14.26 ± 3.07	15.38 ± 3.42
BAI	26.66 ± 2.80	30.74 ± 3.98	29.01 ± 4.07
TYG	8.73 ± 0.64	8.59 ± 0.57	8.65 ± 0.60
VAI	1.45 (0.88, 2.49)	1.55 (1, 2.51)	1.51 (0.95, 2.51)

WC, waist circumference; HC, hip circumference; BMI, body mass index; TG, triglycerides; TC, total cholesterol; HDL-C, high-density lipoprotein cholesterol; SBP, systolic blood pressure; DBP, diastolic blood pressure; FPG, fasting plasma glucose; LAP, lipid accumulation product; BRI, body roundness index; CVAI, Chinese visceral adiposity index; AVI, abdominal volume index; BAI, body adiposity index; TYG, triglyceride glucose; VAI, visceral adiposity index.

### Different characteristics of people with or without MetS

Participants were divided into groups based on sex and whether they had MetS according to the Chinese criteria (2020) (**Table 2**). In men with MetS, the values of the eight obesity- and lipid-related indicators and clinical indicators (SBP, DBP, TG, HDL-C) were significantly increased compared with those of men without MetS (*P* < 0.001). No significant differences in age, height, TC, or eGFR (*P* > 0.05) were noted. In women with MetS, the values of the eight obesity- and lipid-related indicators and clinical indicators (SBP, DBP, TG, HDL-C) were significantly increased compared with those of women without MetS (*P* < 0.001); however, height and TC were not statistically significant (*P* > 0.05). Overall, the values of the eight obesity- and lipid-related indicators and clinical indicators (SBP, DBP, TG, and HDL-C) were significantly increased in participants with MetS compared with those without MetS (*P* < 0.001). TC and eGFR were not significantly different (*P* > 0.05).

**Table 2:** Characteristics of participants with or without MetS (China 2020).

Variable	Male			Female			Total		
	MetS−	MetS+	*P*-value	MetS−	MetS+	*P*-value	MetS−	MetS+	*P*-value
*N*	444	171		703	134		1,147	305	
Age (years)	58.56 ± 14.00	56.82 ± 12.35	0.133	57.03 ± 13.47	64.67 ± 9.79	<0.001	57.62 ± 13.691	60.27 ± 11.93	0.001
Height (cm)	171.03 ± 5.77	171.87 ± 5.13	0.078	159.47 ± 5.53	159.54 ± 5.31	0.889	163.94 ± 7.96	166.45 ± 8.04	<0.001
Weight (kg)	70.73 ± 12.26	78.87 ± 9.51	<0.001	60.56 ± 9.74	67.38 ± 8.36	<0.001	64.50 ± 11.87	73.83 ± 10.49	<0.001
WC (cm)	88.89 ± 8.75	97.56 ± 6.77	<0.001	81.63 ± 8.71	92.61 ± 6.85	<0.001	84.23 ± 9.44	94.57 ± 7.49	<0.001
HC (cm)	98.83 ± 5.75	102.96 ± 5.00	<0.001	97.15 ± 6.47	102.41 ± 6.73	<0.001	97.80 ± 6.25	102.72 ± 5.82	<0.001
BMI (kg/m^2^)	24.13 ± 3.63	26.69 ± 2.79	<0.001	23.84 ± 3.93	26.46 ± 2.92	<0.001	23.95 ± 3.82	26.59 ± 2.85	<0.001
TG (mmol/L)	1.15 (0.85, 1.51)	2.16 (1.81, 2.95)	<0.001	1.15 (0.88, 1.52)	1.9 (1.51, 2.42)	<0.001	1.15 (0.87, 1.52)	2.02 (1.72, 2.8)	<0.001
TC (mmol/L)	4.59 ± 0.95	4.73 ± 0.86	0.066	4.81 ± 0.93	4.98 ± 0.98	0.066	4.72 ± 0.94	4.84 ± 0.92	0.058
HDL-C (mmol/L)	1.41 ± 0.32	1.05 ± 0.23	<0.001	1.58 ± 0.38	1.30 ± 0.31	<0.001	1.51 ± 0.36	1.16 ± 0.29	<0.001
FPG (mmol/L)	5.31 ± 1.23	6.27 ± 2.41	<0.001	5.17 ± 0.98	6.79 ± 2.23	<0.001	5.23 ± 1.08	6.50 ± 2.34	<0.001
SBP (mmHg)	124.43 ± 14.39	132.11 ± 14.10	<0.001	120.69 ± 15.41	142.33 ± 15.35	<0.001	122.14 ± 15.13	136.6 ± 15.49	<0.001
DBP (mmHg)	73.86 ± 9.23	79.56 ± 9.45	<0.001	69.28 ± 9.38	79.49 ± 9.18	<0.001	71.05 ± 9.58	79.52 ± 9.32	<0.001
eGFR (ml/min/1.73 m^2^)	91.78 ± 19.32	91.15 ± 18.16	0.705	89.91 ± 17.52	85.02 ± 14.72	0.003	90.63 ± 18.25	88.46 ± 16.99	0.061
LAP	28.71 (17.18, 40.53)	69.12 (53.36, 109.12)	<0.001	28.75 (17.29, 41.28)	64.94 (49.14, 87.83)	<0.001	28.75 (17.19, 41.04)	67.08 (52.52, 96.51)	<0.001
BRI	3.79 ± 0.98	4.75 ± 0.87	<0.001	3.66 ± 1.15	5.05 ± 1.00	<0.001	3.71 ± 1.09	4.88 ± 0.94	<0.001
CVAI	110.35 (84.13, 133.54)	152.16 (132.38, 170.49)	<0.001	92.27 (64.82, 115.80)	136.13 (122.1, 153.78)	<0.001	98.59 (70.66, 121.51)	142.82 (126.79, 165.71)	<0.001
AVI	16.05 ± 3.07	19.16 ± 2.65	<0.001	13.67 ± 2.81	17.34 ± 2.53	<0.001	14.59 ± 3.13	18.36 ± 2.75	<0.001
BAI	26.24 ± 2.76	27.74 ± 2.59	<0.001	30.33 ± 3.87	32.90 ± 3.88	<0.001	28.74 ± 4.01	30.01 ± 4.11	<0.001
TYG	8.50 ± 0.47	9.35 ± 0.59	<0.001	8.47 ± 0.49	9.22 ± 0.52	<0.001	8.48 ± 0.48	9.29 ± 0.57	<0.001
VAI	1.11 (0.75, 1.61)	2.97 (2.23, 4.49)	<0.001	1.39 (0.93, 2.13)	3.01 (2.13, 4.55)	<0.001	1.29 (0.86, 1.93)	2.99 (2.19, 4.5)	<0.001

WC, waist circumference; HC, hip circumference; BMI, body mass index; TG, triglycerides; TC, total cholesterol; HDL-C, high-density lipoprotein cholesterol; SBP, systolic blood pressure; DBP, diastolic blood pressure; FPG, fasting plasma glucose; LAP, lipid accumulation product; BRI, body roundness index; CVAI, Chinese visceral adiposity index; AVI, abdominal volume index; BAI, body adiposity index; TYG, triglyceride glucose; VAI, visceral adiposity index.

### MetS prevalence and its association with obesity and lipid index

We compared the diagnostic efficacy of MetS with different diagnostic criteria in this population. Under the different criteria, the prevalence of MetS ranged from 21% [China (2020 edition) criteria] to 31.3% (NCEP-ATPIII criteria). We found statistically significant differences in the prevalence of MetS between the China (2020 edition) criteria and the NCEP-ATPIII and IDF criteria, whereas no statistically significant differences were noted between the NCEP-ATPIII and IDF criteria (**Table 3**). Using the China (2020 edition) criteria, the prevalence of MetS among men was significantly greater than that among women (*χ* ^2^ = 29.725, *P* < 0.001), but there was no significant difference in the prevalence of MetS among men and women using the NCEP-ATPIII and IDF standards (*P* > 0.05). In addition, according to multivariate logistic regression analysis, BMI, LAP, BRI, CVAI, AVI, BAI, TYG, and VAI were significantly associated with MetS among all three criteria (*P* < 0.001). After adjusting for age, systolic blood pressure, diastolic blood pressure, TC, and eGFR, the OR value of TYG was 35.069 (22.057–55.757, *P* < 0.001) based on the China (2020 edition) criteria. Using the NCEP-ATPIII criteria, the OR value of TYG was 53.435 (33.535–85.145, *P* < 0.001), and using the IDF criteria, the OR value of TYG was 21.464 (14.726–31.286, *P* < 0.001). Among the three criteria, the LAP group model had the best overall AUC values: China (2020 edition) AUC = 0.925, NCEP-ATPIII criteria AUC = 0.909, and IDF criteria AUC = 0.903 (**Table 4**). The multivariate logistic regression analysis results for the NCEP-ATPIII and IDF standards are shown in **Supplementary Tables 3, 4**. The internal 10-fold cross-validation and penalty regression for validation are shown in **Supplementary Tables 5–13**.

**Table 3:** Prevalence of MetS by different criteria.

Criterion	Male (*n* = 615)			Female (*n* = 837)			Total (*n* = 1,452)		
	MetS−	MetS+	%	MetS−	MetS+	%	MetS−	MetS+	%
China (2020)	444	171	27.8	703	134	16.0^*^	1,147	305	21.0
NCEP-ATPIII	418	197	32.0	579	258	30.8	997	455	31.3^&^
IDF	433	182	29.6	589	248	29.6	1,022	430	29.6^&^

^*^P < 0.05 compared with male patients; ^&^P < 0.05 compared with China (2020).

**Table 4:** Predictive value of the eight obesity- and lipid-related indices in the China (2020) criteria and multivariate logistic regression analysis.

	Male			Female			All		
	OR	*P*	AUC	OR	*P*	AUC	OR	*P*	AUC
Index	1.251 (1.171–1.337)	<0.001	0.781	1.152 (1.085–1.223)	<0.001	0.874	1.209 (1.157–1.263)	<0.001	0.822
LAP	1.063 (1.050–1.075)	<0.001	0.915	1.060 (1.048–1.073)	<0.001	0.939	1.062 (1.054–1.071)	<0.001	0.925
BRI	2.748 (2.181–3.462)	<0.001	0.809	2.378 (1.914–2.955)	<0.001	0.901	2.57 (2.206–2.993)	<0.001	0.853
CVAI	1.041 (1.033–1.050)	<0.001	0.869	1.051 (1.039–1.063)	<0.001	0.924	1.044 (1.038–1.05)	<0.001	0.902
AVI	1.407 (1.305–1.518)	<0.001	0.820	1.485 (1.359–1.624)	<0.001	0.908	1.451 (1.374–1.532)	<0.001	0.871
BAI	1.196 (1.114–1.284)	<0.001	0.732	1.119 (1.061–1.180)	<0.001	0.862	1.065 (1.03–1.101)	<0.001	0.775
TYG	45.563 (22.641–91.690)	<0.001	0.911	26.128 (13.651–50.012)	<0.001	0.935	35.069(22.057–55.757)	<0.001	0.919
VAI	4.025 (3.104–5.218)	<0.001	0.916	2.044 (1.732–2.411)	<0.001	0.916	2.445 (2.145–2.788)	<0.001	0.895

Adjusted factors: systolic blood pressure, diastolic blood pressure, total cholesterol, and eGFR.

BMI, body mass index; LAP, lipid accumulation product; BRI, body roundness index; CVAI, Chinese visceral adiposity index; AVI, abdominal volume index; BAI, body adiposity index; TYG, triglyceride glucose; VAI, visceral adiposity index.

### Receiver operating characteristic analysis

**Figure 1:**
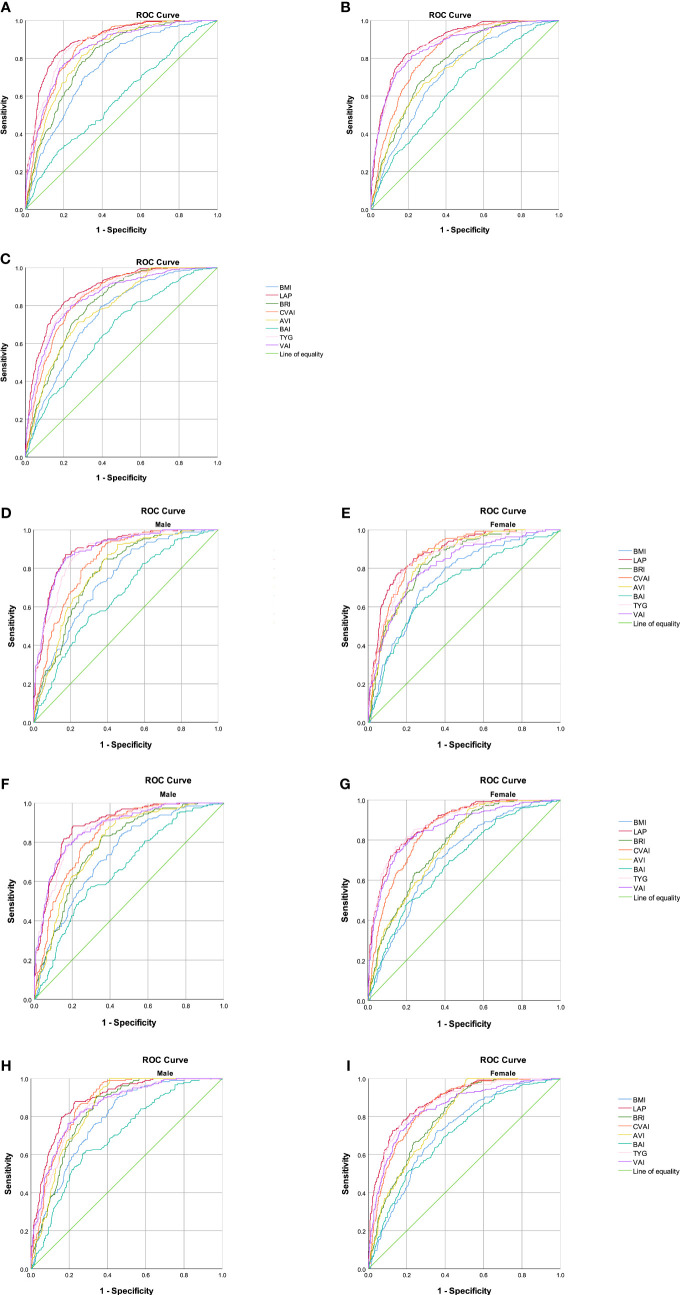
Comparison of the diagnostic values of BMI, LAP, BRI, CVAI, BAI, AVI, TYG, and VAI in predicting metabolic syndrome using three criteria in a relatively healthy Chinese population. **(A)** Chinese (2020 edition) criteria; **(B)** NCEP-ATPIII criteria; **(C)** IDF criteria. Chinese (2020) criteria: **(D)** obesity- and lipid-related indices for a relatively healthy Chinese population, men; **(E)** obesity- and lipid-related indices for a relatively healthy Chinese population, women. NCEP-ATPIII criteria: **(F)** obesity- and lipid-related indices for a relatively healthy Chinese population, men; **(G)** obesity- and lipid-related indices for a relatively healthy Chinese population, women. IDF criteria: **(H)** obesity- and lipid-related indices for a relatively healthy Chinese population, men; **(I)** obesity- and lipid-related indices for a relatively healthy Chinese population, women.

The abilities of BMI, LAP, BRI, CVAI, AVI, BAI, VAI, and TYG to predict MetS were analyzed by ROC curves based on the different criteria (**Table 5**). We found that LAP had the highest AUC values of 0.893 (0.874–0.912), 0.886 (0.869–0.903), and 0.882 (0.864–0.899) for the three diagnostic criteria. Second, the AUC values of CVAI, TYG, and VAI were all greater than 0.8. The subgroup analysis based on sex found that LAP had the highest AUC value for all three diagnostic criteria followed by CVAI, TYG, and VAI (**Figure 1**). Using the Guidelines for the Prevention and Treatment of Type 2 Diabetes in China (2020 edition) as the diagnostic criteria for MetS, in men, the AUC of LAP was the greatest at 0.90 (0.874–0.926) followed by VAI (AUC = 0.896). In women, LAP exhibited the greatest AUC of 0.882 (0.853–0.911) followed by CVAI (AUC = 0.870). Using NCEP-ATPIII as the diagnostic criteria, LAP exhibited the greatest AUC of 0.889 (0.863–0.915) followed by VAI (AUC = 0.875) in men. In women, LAP exhibited the greatest AUC of 0.885 (0.862 to 0.908) followed by TYG (AUC = 0.883). Using IDF as the diagnostic criteria, in men, LAP exhibited the greatest AUC of 0.884 (0.857 to 0.911) followed by CVAI (AUC = 0.868). In women, LAP exhibited the greatest AUC of 0.883 (0.860–0.906) followed by TYG (AUC = 0.866). A pairwise comparison of the AUC values for predicting MetS using the eight indicators based on the three criteria found that the AUC values for LAP were higher than those of the other seven indices, and the difference was statistically significant (*P* < 0.05). However, no statistically significant differences were noted between LAP and TYG using the NCEP-ATPIII criteria. Moreover, we found that BRI had the best forecasting ability for MetS among the four anthropometric indicators (BMI, BRI, AVI, and BAI), and the difference was statistically significant (*P* < 0.05) (**Supplementary Tables 16–18**). The optimal cutoff values of the eight obesity- and lipid-related indicators for predicting MetS in the three sets of criteria are displayed in **Table 6** and **Supplementary Tables 14, 15**.

**Table 5:** Area under the curve of seven obesity- and lipid-related indices with the different metabolic syndrome criteria.

Group	Variable	MetS-China (2020) criterion		MetS-NCEP-ATPIII criterion		MetS-IDF criterion	
		AUC (95% CI)	*P*-value	AUC (95% CI)	*P*-value	AUC (95% CI)	*P*-value
All	BMI	0.75 (0.722–0.779)	<0.001	0.722 (0.695–0.749)	<0.001	0.745 (0.719–0.771)	<0.001
	LAP	0.893 (0.874–0.912)	<0.001	0.886 (0.869–0.903)	<0.001	0.882 (0.864–0.899)	<0.001
	BRI	0.804 (0.779–0.829)	<0.001	0.775 (0.751–0.799)	<0.001	0.805 (0.783–0.828)	<0.001
	CVAI	0.86 (0.84–0.88)	<0.001	0.832 (0.811–0.853)	<0.001	0.845 (0.825–0.865)	<0.001
	AVI	0.823 (0.80–0.847)	<0.001	0.756 (0.731–0.782)	<0.001	0.781 (0.757–0.805)	<0.001
	BAI	0.587 (0.55–0.623)	<0.001	0.641 (0.611–0.671)	<0.001	0.665 (0.635–0.695)	<0.001
	TYG	0.874 (0.853–0.895)	<0.001	0.877 (0.858–0.895)	<0.001	0.854 (0.833–0.874)	<0.001
	VAI	0.849 (0.825–0.872)	<0.001	0.864 (0.844–0.885)	<0.001	0.845 (0.823–0.867)	<0.001
Male	BMI	0.742 (0.701–0.783)	<0.001	0.739 (0.699–0.779)	<0.001	0.781 (0.744–0.818)	<0.001
	LAP	0.90 (0.874–0.926)	<0.001	0.889 (0.863–0.915)	<0.001	0.884 (0.857–0.911)	<0.001
	BRI	0.777 (0.740–0.815)	<0.001	0.782 (0.746–0.819)	<0.001	0.827 (0.795–0.859)	<0.001
	CVAI	0.835 (0.803–0.866)	<0.001	0.836 (0.805–0.867)	<0.001	0.868 (0.841–0.895)	<0.001
	AVI	0.788 (0.751–0.825)	<0.001	0.793 (0.757–0.829)	<0.001	0.838 (0.808–0.868)	<0.001
	BAI	0.659 (0.613–0.706)	<0.001	0.665 (0.620–0.709)	<0.001	0.699 (0.655–0.742)	<0.001
	TYG	0.886 (0.859–0.913)	<0.001	0.872 (0.843–0.90)	<0.001	0.841 (0.809–0.873)	<0.001
	VAI	0.896 (0.870–0.923)	<0.001	0.875 (0.846–0.904)	<0.001	0.850 (0.819–0.882)	<0.001
Female	BMI	0.749 (0.707–0.792)	<0.001	0.711 (0.675–0.747)	<0.001	0.722 (0.687–0.758)	<0.001
	LAP	0.882 (0.853–0.911)	<0.001	0.885 (0.862–0.908)	<0.001	0.883 (0.860–0.906)	<0.001
	BRI	0.830 (0.796–0.864)	<0.001	0.775 (0.743–0.806)	<0.001	0.797 (0.767–0.827)	<0.001
	CVAI	0.870 (0.842–0.898)	<0.001	0.849 (0.824–0.875)	<0.001	0.854 (0.828–0.88)	<0.001
	AVI	0.842 (0.810–0.873)	<0.001	0.771 (0.739–0.802)	<0.001	0.794 (0.764–0.823)	<0.001
	BAI	0.710 (0.661–0.758)	<0.001	0.689 (0.651–0.727)	<0.001	0.705 (0.668–0.742)	<0.001
	TYG	0.858 (0.825–0.891)	<0.001	0.883 (0.859–0.907)	<0.001	0.866 (0.840–0.892)	<0.001
	VAI	0.815 (0.776–0.854)	<0.001	0.859 (0.831–0.887)	<0.001	0.843 (0.814–0.873)	<0.001

BMI, body mass index; LAP, lipid accumulation product; BRI, body roundness index; CVAI, Chinese visceral adiposity index; AVI, abdominal volume index; BAI, body adiposity index; TYG, triglyceride glucose; VAI, visceral adiposity index.

**Table 6:** The cutoff, sensitivities, specificities, and Youden’s index of each variable for the screening of metabolic syndrome in the China (2020) criteria.

Group	Variable	Optimal cutoff values	Youden’s index	Sensitivity (%)	Specificity (%)
All	BMI	24.10	0.396	82.6	57.0
	LAP	46.3	0.656	83.6	82.0
	BRI	3.99	0.489	84.9	64.0
	CVAI	119.06	0.595	86.9	72.6
	AVI	16.02	0.522	81.3	70.9
	BAI	31.91	0.137	31.1	82.6
	TYG	8.83	0.629	84.3	78.6
	VAI	2.23	0.569	74.8	82.1
Male	BMI	24.01	0.374	85.4	52.0
	LAP	46.28	0.698	87.1	82.7
	BRI	3.99	0.464	83.6	62.8
	CVAI	119.06	0.540	91.2	62.8
	AVI	16.23	0.469	92.4	54.5
	BAI	27.30	0.25	55.6	69.4
	TYG	8.85	0.645	85.4	79.1
	VAI	1.77	0.681	85.4	82.7
Female	BMI	25.35	0.417	68.7	73.0
	LAP	47.02	0.62	79.1	82.9
	BRI	4.21	0.544	82.1	72.3
	CVAI	114.39	0.614	87.3	74.1
	AVI	15.14	0.561	82.1	74
	BAI	32.02	0.356	61.2	74.4
	TYG	8.81	0.608	82.8	78.0
	VAI	2.24	0.518	73.1	78.7

BMI, body mass index; LAP, lipid accumulation product; BRI, body roundness index; CVAI, Chinese visceral adiposity index; AVI, abdominal volume index; BAI, body adiposity index; TYG, triglyceride glucose; VAI, visceral adiposity index.

## Discussion

Given the economic development and lifestyle changes, the prevalence of MetS is increasing worldwide and has become an important public health issue (28). In developed countries, such as the United States, the prevalence rate of MetS is 34.7% according to the National Health and Nutrition Examination Survey (2011–2016) (29). In China, the largest developing country, the prevalence of MetS has shown an increasing tendency. Analysis of China Nutrition and Health Surveillance data (2015–2017) found that the prevalence of metabolic syndrome among residents aged 20 years and older was 31.1% (30). The MetS diagnostic criteria are also being modified and improved, and the IDF and NECP-ATPIII criteria are the most widely used worldwide. Due to differences among ethnic groups, China has developed criteria for the diagnosis of MetS. In the MetS diagnostic criteria of the Chinese Guidelines for the Prevention and Treatment of Type 2 Diabetes (2020 edition), the cutoff points of WC, HDL-C, and FPG are different from those of the IDF and NECP-ATPIII criteria. This difference may explain why the prevalence of MetS found in this study with the China (2020 edition) criteria is lower than that with the NCEP-ATPIII and IDF criteria.

In this study, we investigated the ability of the eight obesity- and lipid-related indicators, namely, BMI, LAP, BRI, CVAI, BAI, AVI, TYG, and VAI, to predict MetS in relatively healthy people under different diagnostic criteria. We found that these eight obesity- and lipid-related indicators had reliable predictive value for MetS. Furthermore, LAP outperformed the other seven parameters in predicting MetS. Following the model design, it was discovered that the best logistic models were those using LAP, age, SBP, DBP, TC, and eGFR, which is consistent with our practice of utilizing a sole indicator to forecast MetS. Therefore, we conclude that LAP is superior for predicting MetS in relatively healthy Chinese adults. These results demonstrate that LAP is a simple and powerful tool for clinical use. This is the first study to assess the ability of these eight obesity- and lipid-related indicators to predict MetS in a relatively healthy population under different diagnostic criteria.

BMI, BRI, AVI, and BAI are all calculated based on anthropometric measurements, and our results show that these indicators are closely related to MetS. BMI has been shown to be a risk factor for various cardiovascular and metabolic diseases and mortality (31), but it cannot distinguish between subcutaneous and visceral fat (6, 32). BRI is a novel obesity-related index that uses WC and height to estimate body fat and visceral adipose tissue (7). Rico-Martin et al. (11) found that BRI was a better predictor of MetS among different ethnic and racial groups than BMI. This finding is consistent with our study, where we found that BRI has better predictive power for MetS than the other three anthropometric constructs (BMI, AVI, BAI). The AUC of BRI for women with MetS can be as high as 0.83 using the China (2020 edition) standards. In addition, AVI is calculated using the total abdominal volume assessment from the symphysis pubis to the xiphoid process to reflect visceral fat content. Perona et al. (15) found that WC and AVI had a strong ability to predict MetS in adolescents when using the IDF criteria. Wu et al. (33) found that AVI had good performance in identifying MetS in non-overweight/obese Chinese adults (men, 0.743; women, 0.819), which is similar to our results with the Chinese (2020 edition) criteria (men, 0.775; women, 0.831). The BAI also showed some predictive ability for MetS in a Colombian population and among Chinese postmenopausal women (34, 35). In our study, BAI was relatively weak in predicting MetS with an AUC less than 0.8, which may be due to different ethnic groups and population characteristics. Although BMI, BRI, AVI, and BAI can all predict MetS, the combination of anthropometric values and lipid-related indicators exhibited a better ability to predict MetS in our study.

LAP, CVAI, VAI, and TYG are new proxies for central obesity and lipid accumulation and can be used to assess visceral fat distribution and reflect visceral fat dysfunction by combining anthropometric markers with lipid or glucose markers. In this study, we found that the AUC values of LAP, CVAI, VAI, and TYG for the three sets of criteria were all greater than 0.8, showing good predictive performance. Since Kahn (24) proposed the LAP, several studies have found that LAP has a good ability to predict MetS (36–38), and it is calculated based on sex to better reflect the relationships between fat accumulation and lipid toxicity and cardiac metabolic disease (39). Guo et al. (40) compared the ability of LAP, VAI, BAI, and WHtR to predict MetS in low-income rural adults in Xinjiang, China, and found that LAP was a better indicator to predict MetS than the other three factors. In a Brazilian population free of cardiovascular disease and type 2 diabetes, LAP had a reliable diagnostic value for MetS compared with classic anthropometric measures (BMI, WC, waist-to-height ratio, waist-to-hip ratio) when using the American Heart Association (AHA)/National Heart, Lung and Blood Institute (NHLBI), IDF, and harmonized AHA/NHLBI and IDF standards (41). In a cross-sectional study of 552 healthy Argentine men, the AUC for LAP in predicting MetS was 0.91 (42). Our study also showed that LAP had the strongest predictive ability for MetS with a maximum AUC of 0.90. These results underscore the importance of LAP in predicting MetS in clinical practice. Xia et al. (17) believe that CVAI is a reliable and applicable indicator for evaluating visceral fat dysfunction in Chinese people and even for evaluating the metabolic health status of Asian people. Our study shows that CVAI also has a good ability to predict MetS with AUC values greater than 0.8 for all three criteria. In addition, VAI reflects abdominal fat distribution and dyslipidemia and is associated with insulin resistance (IR), abnormal glucose balance, and an increased risk of cardiovascular disease in adults (43, 44). Our previous study in patients with chronic kidney disease found that VAI had a good ability to predict MetS (45), which is consistent with the findings of this study. TYG, a product of TG and FPG, is a new visceral fat assessment tool that is associated with IR (46, 47). A Chinese study also confirmed the ability of the TYG index to identify metabolically unhealthy Chinese adults and those at high risk of cardiovascular and metabolic diseases (48). Lee et al. (49) found that TYG was a good predictor of MetS in metabolically obese but normal-weight individuals in Korea with an AUC between 0.855 and 0.868. In our study, TYG also had excellent predictive ability with an AUC between 0.841 and 0.886. The occurrence of MetS in central obesity may be closely related to the increase in visceral adipose tissue, the decrease in subcutaneous tissue expansion, and the metabolic changes in triglycerides stored in different organs, which may explain the better predictive ability of the four indicators for MetS (50).

The study had several limitations. First, this study was a cross-sectional study with a limited sample size, and we could not determine the causal relationships. Second, the survey included only individuals belonging to the relatively healthy population in China, so caution should be taken when generalizing the results to other races and groups. Third, the study did not document details about long-term medication use, education, or health status, which may have influenced the results. Finally, this is a cross-sectional study of the relatively healthy population in a community with an imbalanced proportion of controls and patients.

## Conclusion

Our study shows that using different criteria, LAP, TYG, CVAI, and VAI have significant predictive efficacy for MetS in a relatively healthy population in China. LAP exhibits the best predictive efficacy, regardless of sex.

## Data availability statement

The original contributions presented in the study are included in the article/**Supplementary Material**. Further inquiries can be directed to the corresponding authors.

## Ethics statement

The studies involving human participants were reviewed and approved by the Ethics Committee of the Chinese People’s Liberation Army General Hospital. The patients/participants provided their written informed consent to participate in this study.

## Author contributions

HJ and XmC, conceptualization. YD and WZ, visualization. YC, XL, ZD and YZ, funding acquisition. YD and WZ, formal analysis. ZF, YW, DZ, XS, XzC, and GC, resources. YD and WZ, writing—original draft preparation. ZL, YN and YZ, writing–review and editing. QL, HP and HL, supervision. All authors contributed to the article and approved the submitted version.

## Funding

Part of this research was funded by the National Key Research and Development Program of China (2022YFC3602900), the Innovation Platform for Academicians of Hainan Province (Academician Chen Xiangmei of Hainan Province Kidney Diseases Team Innovation Center), the Specialized Scientific Program of the Innovation Platform for Academicians of Hainan Province (YSPTZX202026), the Specialized Scientific Research Project of Military Health Care (21BJZ37), the National Natural Science Foundation of China (82030025), and the Clinical Research Support Fund, Young Talent Project, Chinese PLA General Hospital (2019XXMBD-005, 2019XXJSYX01), Construction Project of Improving Medical Service Capacity of Provincial Medical Institutions in Henan Province (2017), Medical and Health Research Project in Luoyang (2001027A).

## Acknowledgments

We wish to thank the study participants for their cooperation and participation.

## Conflict of interest

The authors declare that the research was conducted in the absence of any commercial or financial relationships that could be construed as a potential conflict of interest.

## Publisher’s note

All claims expressed in this article are solely those of the authors and do not necessarily represent those of their affiliated organizations, or those of the publisher, the editors and the reviewers. Any product that may be evaluated in this article, or claim that may be made by its manufacturer, is not guaranteed or endorsed by the publisher.

## Abbreviations

LAP, lipid accumulation product; BRI, body roundness index; CVAI: Chinese visceral adiposity index; AVI, abdominal volume index; BAI, body adiposity index; TYG, triglyceride glucose; VAI, visceral adiposity index.
