# Supplementary material for: Predictive ability of obesity- and lipid-related indicators for metabolic syndrome in relatively healthy Chinese adults
Source: Front Endocrinol (Lausanne). 2022 Nov 18;13:1016581. doi: 10.3389/fendo.2022.1016581 (PMC9715593; doi:10.3389/fendo.2022.1016581)
Supplement: Supplementary file 1 [file DataSheet_1.docx]

**Predictive ability of obesity- and lipid-related indicators for metabolic syndrome in relatively healthy Chinese adults**

Given economic development and lifestyle changes, the prevalence of metabolic syndrome (MetS) is increasing worldwide and has become an important public health issue. Therefore, early screening and early diagnosis of MetS are of great importance.This is the first study to assess the ability of these eight obesity- and lipid-related indicators [body mass index (BMI), lipid accumulation product (LAP), body roundness index (BRI), Chinese visceral adiposity index (CVAI), body adiposity index (BAI), abdominal volume index (AVI), triglyceride glucose index (TYG), and visceral adiposity index (VAI)]to predict MetS in relatively healthy population under different diagnostic criteria.We found that LAP, TYG, CVAI and VAI are of high value for MetS prediction in relatively healthy population in China. LAP the best predictor of MetS in relatively healthy Chinese population.

This is the first study to discover that LAP is of excellent value for MetS prediction compared to other obesity- and lipid-related indicators (BMI, BRI, CVAI, BAI, AVI, TYG, VAI) under different diagnostic criteria. We recommend modifying the lifestyle to stay healthy through early LAP index monitoring in relatively healthy Chinese population.
