# Supplementary material for: Predictive ability of obesity- and lipid-related indicators for metabolic syndrome in relatively healthy Chinese adults
Source: Front Endocrinol (Lausanne). 2022 Nov 18;13:1016581. doi: 10.3389/fendo.2022.1016581 (PMC9715593; doi:10.3389/fendo.2022.1016581)
Supplement: Supplementary file 2 [file DataSheet_2.docx]

Supplementary Material

# Supplementary Tables

**Supplementary Table 1.** Formulas for calculating eight obesity- and lipid-related indices.

| Index | Equation |
| --- | --- |
| BMI | BMI = BW _kg_ / BH _m_^2^ |
| LAP | Male: LAP = (WC _cm_ - 65) × TG _mmol/L_ |
|  | Female: LAP = (WC _cm_ - 58) × TG _mmol/L_ |
| BRI | BRI = 364.2 − 365.5 × [1 - (WC _cm_/2π)^2^/ (0.5 * BH _cm_) ^2^]^0.5^ |
| CVAI | Male: CVAI = -267.93 + 0.68 × age _y_ + 0.03 × BMI _kg/m2_ + 4.00 × WC _cm_ + 22.00 × Lg TG _mmol/L_- 16.32 × HDL _mmol/L_ |
|  | Female: CVAI = -187.32 + 1.71 × age _y_ + 4.32 × BMI _kg/m2_ + 1.12 × WC _cm_ + 39.76 × Lg TG _mmol/L_- 11.66 × HDL _mmol/L_ |
| AVI | AVI = [2× WC_cm_^2^ + 0.7 × (WC _cm_ - HC _cm_)^2^]/1000 |
| BAI | BAI = HC _cm_ / BH _m_^3/2^ -18 |
| TYG | TYG = ln [FTG _mg/dl_ × (FPG _mg/dl_ / 2)] |

BMI, Body mass index; LAP, Lipid accumulation product; BRI, Body roundness index; CVAI, Chinese visceral adiposity index; AVI, Abdominal volume index; BAI, Body adiposity index; TYG, Triglyceride glucose; VAI, Visceral adiposity index; BW: Body weight; BH: Body height; WC, Waist circumference; HC, Hip circumference; TG, Triglycerides; HDL-C, High-density lipoprotein cholesterol; FTG: Fasting triglycerides; FPG: Fasting plasma glucose.

**Supplementary Table 2.** Definition of MetS

| Variables | Recommended thresholds | | |
| --- | --- | --- | --- |
|  | China (2020) Criterion | NCEP-ATPIII Criterion | IDF Criterion |
| Abdominal obesity  (waist circumference) | ≥ 90 cm (male);  ≥ 85 cm (female) | ≥ 90 cm (male);  ≥ 80 cm (female) | ≥ 90 cm (male);  ≥ 80 cm (female) |
| Triglycerides | ≥ 1.7 mmol/L | ≥ 1.7 mmol/L | ≥ 1.7 mmol/L or treatment |
| Plasma high-density  lipoprotein cholesterol | < 1.04 mmol/L | < 1.03 mmol/L (male);  < 1.29 mmol/L (female) or treatment | < 1.03 mmol/L (male);  < 1.29 mmol/L (female) or treatment |
| Blood pressure | Systolic ≥ 130 or diastolic ≥ 85 mmHg and (or) previously diagnosed hypertension or treatment | Systolic ≥ 130 or diastolic ≥ 85 mmHg and (or) previously diagnosed hypertension or treatment | Systolic ≥ 130 or diastolic  ≥ 85 mmHg and (or) previously diagnosed hypertension or treatment |
| Fasting plasma glucose | ≥ 6.1 mmol/L or 2hPG≥ 7.8 mmol/L and (or) previously diagnosed DM or treatment | ≥ 5.6 mmol/L and (or) previously diagnosed DM or treatment | ≥ 5.6 mmol/L and (or) previously  diagnosed T2DM or treatment |
| Diagnosed conditions | Three or more can be considered MetS | Three or more can be considered MetS | Abdominal obesity + Any two or more |

**Supplementary Table 3.** Predictive value of eight obesity- and lipid-related indices with the NCEP-ATPIII criteria and multivariate logistic regression analysis.

|  | male | | | female | | | all | | |
| --- | --- | --- | --- | --- | --- | --- | --- | --- | --- |
|  | OR | P | AUC | OR | P | AUC | OR | P | AUC |
| Index | 1.252 (1.174-1.336) | <0.001 | 0.777 | 1.149 (1.091-1.210) | <0.001 | 0.812 | 1.2 (1.153-1.249) | <0.001 | 0.787 |
| LAP | 1.064 (1.052-1.077) | <0.001 | 0.906 | 1.081 (1.067-1.096) | <0.001 | 0.916 | 1.071 (1.062-1.08) | <0.001 | 0.909 |
| BRI | 2.883 (2.291-3.627) | <0.001 | 0.813 | 1.991 (1.680-2.359) | <0.001 | 0.827 | 2.367 (2.068-2.709) | <0.001 | 0.813 |
| CVAI | 1.042 (1.034-1.050) | <0.001 | 0.869 | 1.051 (1.041-1.060) | <0.001 | 0.881 | 1.038 (1.033-1.043) | <0.001 | 0.863 |
| AVI | 1.440 (1.334-1.554) | <0.001 | 0.824 | 1.319 (1.235-1.409) | <0.001 | 0.828 | 1.318 (1.262-1.377) | <0.001 | 0.808 |
| BAI | 1.199 (1.120-1.284) | <0.001 | 0.730 | 1.138 (1.084-1.194) | <0.001 | 0.803 | 1.121 (1.085-1.158) | <0.001 | 0.758 |
| TYG | 40.665 (20.999-78.749) | <0.001 | 0.897 | 74.961 (37.678-149.136) | <0.001 | 0.925 | 53.435 (33.535-85.145) | <0.001 | 0.911 |
| VAI | 4.104 (3.166-5.321) | <0.001 | 0.896 | 3.757 (3.009-4.691) | <0.001 | 0.916 | 3.933 (3.327-4.649) | <0.001 | 0.904 |

BMI, Body mass index; LAP, Lipid accumulation product; BRI, Body roundness index; CVAI, Chinese visceral adiposity index; AVI, Abdominal volume index; BAI, Body adiposity index; TYG, Triglyceride glucose; VAI, Visceral adiposity index. Adjusted factors: systolic blood pressure, diastolic blood pressure, total cholesterol and eGFR.

**Supplementary Table 4.** Predictive value of eight obesity- and lipid-related indices with the IDF criteria and multivariate logistic regression analysis.

|  | male | | | female | | | all | | |
| --- | --- | --- | --- | --- | --- | --- | --- | --- | --- |
|  | OR | P | AUC | OR | P | AUC | OR | P | AUC |
| Index | 1.322 (1.232-1.418) | <0.001 | 0.798 | 1.165 (1.105-1.229) | <0.001 | 0.813 | 1.235 (1.184-1.287) | <0.001 | 0.793 |
| LAP | 1.054 (1.044-1.065) | <0.001 | 0.895 | 1.080 (1.066-1.094) | <0.001 | 0.914 | 1.065 (1.057-1.074) | <0.001 | 0.903 |
| BRI | 3.637 (2.819-4.693) | <0.001 | 0.842 | 2.218 (1.857-2.650) | <0.001 | 0.837 | 2.742 (2.374-3.167) | <0.001 | 0.828 |
| CVAI | 1.049 (1.040-1.058) | <0.001 | 0.890 | 1.053 (1.043-1.063) | <0.001 | 0.884 | 1.041 (1.036-1.046) | <0.001 | 0.868 |
| AVI | 1.557 (1.430-1.696) | <0.001 | 0.855 | 1.375 (1.283-1.474) | <0.001 | 0.838 | 1.37 (1.308-1.435) | <0.001 | 0.818 |
| BAI | 1.263 (1.176-1.358) | <0.001 | 0.739 | 1.160 (1.104-1.219) | <0.001 | 0.806 | 1.15 (1.113-1.189) | <0.001 | 0.758 |
| TYG | 14.283 (8.598-23.726) | <0.001 | 0.863 | 34.145 (19.087-61.083) | <0.001 | 0.910 | 21.464 (14.726-31.286) | <0.001 | 0.888 |
| VAI | 2.52 (2.076-3.060) | <0.001 | 0.869 | 3.001 (2.475-3.664) | <0.001 | 0.901 | 2.804 (2.444-3.216) | <0.001 | 0.883 |

BMI, Body mass index; LAP, Lipid accumulation product; BRI, Body roundness index; CVAI, Chinese visceral adiposity index; AVI, Abdominal volume index; BAI, Body adiposity index; TYG, Triglyceride glucose; VAI, Visceral adiposity index. Adjusted factors: systolic blood pressure, diastolic blood pressure, total cholesterol and eGFR.

**Supplementary Table 5.** The result of mean validation AUCs of different models in 10-fold cross-validation in the China (2020) criteria, both sex groups.

|  | BMI | LAP | BRI | CVAI | AVI | BAI | TYG | VAI |
| --- | --- | --- | --- | --- | --- | --- | --- | --- |
| Base | 0.815799 | 0.921133 | 0.847844 | 0.900206 | 0.866712 | 0.77001 | 0.915213 | 0.891078 |
| Ridge (a=0) | 0.815775 | 0.915438 | 0.847943 | 0.896084 | 0.865962 | 0.770559 | 0.914311 | 0.887259 |
| Elasticnet a=0.1 | 0.81681 | 0.920721 | 0.848167 | 0.900339 | 0.867213 | 0.776243 | 0.915412 | 0.890933 |
| Elasticnet a=0.2 | 0.817325 | 0.920754 | 0.848124 | 0.900361 | 0.867602 | 0.775841 | 0.915697 | 0.890845 |
| Elasticnet a=0.3 | 0.817704 | 0.92083 | 0.848096 | 0.90039 | 0.868206 | 0.775191 | 0.91609 | 0.89078 |
| Elasticnet a=0.4 | 0.817885 | 0.920927 | 0.848303 | 0.900358 | 0.868571 | 0.775041 | 0.916033 | 0.890826 |
| Elasticnet a=0.5 | 0.81836 | 0.920984 | 0.84855 | 0.900411 | 0.868864 | 0.77491 | 0.916228 | 0.890719 |
| Elasticnet a=0.6 | 0.818753 | 0.921072 | 0.848607 | 0.900386 | 0.869112 | 0.774729 | 0.916302 | 0.890775 |
| Elasticnet a=0.7 | 0.818813 | 0.921078 | 0.84835 | 0.90045 | 0.869224 | 0.774519 | 0.916435 | 0.890697 |
| Elasticnet a=0.8 | 0.818754 | 0.921413 | 0.848259 | 0.900428 | 0.869398 | 0.774299 | 0.916401 | 0.890782 |
| Elasticnet a=0.9 | 0.818748 | 0.92143 | 0.84823 | 0.900443 | 0.869325 | 0.77419 | 0.916388 | 0.890856 |
| Lasso (a=1) | 0.818649 | 0.92154 | 0.848252 | 0.90044 | 0.869491 | 0.774241 | 0.916298 | 0.890898 |
| **best model** | **a=0.7 L=0.013** | **a=1 L=0.003** | **a=0.6 L=0.052** | **a=0.7 L=0.003** | **a=1 L=0.022** | **a=0.1 L=0.229** | **a=0.7 L=0.005** | **Base** |
| **best result** | **0.818813** | **0.92154** | **0.848607** | **0.90045** | **0.869491** | **0.776243** | **0.916435** | **0.891078** |

BMI, Body mass index; LAP, Lipid accumulation product; BRI, Body roundness index; CVAI, Chinese visceral adiposity index; AVI, Abdominal volume index; BAI, Body adiposity index; TYG, Triglyceride glucose; VAI, Visceral adiposity index; L, lambda.

**Supplementary Table 6.** The result of mean validation AUCs of different models in 10-fold cross-validation in the China (2020) criteria, male group.

|  | BMI | LAP | BRI | CVAI | AVI | BAI | TYG | VAI |
| --- | --- | --- | --- | --- | --- | --- | --- | --- |
| Base | 0.778705 | 0.91939 | 0.805688 | 0.866517 | 0.814755 | 0.728151 | 0.912848 | 0.916489 |
| Ridge (a=0) | 0.779252 | 0.90595 | 0.808481 | 0.866876 | 0.815382 | 0.732443 | 0.909207 | 0.903706 |
| Elasticnet a=0.1 | 0.779571 | 0.918892 | 0.811713 | 0.867553 | 0.818115 | 0.734155 | 0.912965 | 0.91665 |
| Elasticnet a=0.2 | 0.781547 | 0.919027 | 0.809796 | 0.867685 | 0.816768 | 0.735051 | 0.913205 | 0.91704 |
| Elasticnet a=0.3 | 0.781771 | 0.919136 | 0.810087 | 0.868032 | 0.816803 | 0.734722 | 0.913491 | 0.91704 |
| Elasticnet a=0.4 | 0.782194 | 0.919136 | 0.810256 | 0.868265 | 0.81598 | 0.734536 | 0.91335 | 0.917211 |
| Elasticnet a=0.5 | 0.78226 | 0.919136 | 0.810357 | 0.868548 | 0.81584 | 0.734299 | 0.913381 | 0.917338 |
| Elasticnet a=0.6 | 0.782045 | 0.919136 | 0.80995 | 0.868687 | 0.815729 | 0.733868 | 0.9134 | 0.918085 |
| Elasticnet a=0.7 | 0.781926 | 0.920986 | 0.80952 | 0.868667 | 0.815814 | 0.732873 | 0.913606 | 0.918958 |
| Elasticnet a=0.8 | 0.781734 | 0.922421 | 0.809284 | 0.868667 | 0.815617 | 0.733107 | 0.913316 | 0.920157 |
| Elasticnet a=0.9 | 0.781639 | 0.922256 | 0.80916 | 0.868539 | 0.815736 | 0.732854 | 0.914201 | 0.921577 |
| Lasso (a=1) | 0.781735 | 0.922984 | 0.809128 | 0.86841 | 0.815803 | 0.732749 | 0.914651 | 0.922057 |
| **best model** | **a=0.5 L=0.019** | **a=1 L=0.056** | **a=0.1 L=0.419** | **a=0.6 L=0.013** | **a=0.1 L=0.473** | **a=0.2 L=0.064** | **a=1 L=0.016** | **a=1 L=0.054** |
| **best result** | **0.78226** | **0.922984** | **0.811713** | **0.868687** | **0.818115** | **0.735051** | **0.914651** | **0.922057** |

BMI, Body mass index; LAP, Lipid accumulation product; BRI, Body roundness index; CVAI, Chinese visceral adiposity index; AVI, Abdominal volume index; BAI, Body adiposity index; TYG, Triglyceride glucose; VAI, Visceral adiposity index; L, lambda.

**Supplementary Table 7.** The result of mean validation AUCs of different models in 10-fold cross-validation in the China (2020) criteria, female group.

|  | BMI | LAP | BRI | CVAI | AVI | BAI | TYG | VAI |
| --- | --- | --- | --- | --- | --- | --- | --- | --- |
| Base | 0.870303 | 0.931074 | 0.900409 | 0.91794 | 0.906641 | 0.856045 | 0.926885 | 0.909223 |
| Ridge (a=0) | 0.870917 | 0.931853 | 0.901112 | 0.917003 | 0.907367 | 0.857447 | 0.926882 | 0.909801 |
| Elasticnet a=0.1 | 0.871091 | 0.932463 | 0.901303 | 0.918543 | 0.907524 | 0.858805 | 0.928239 | 0.909981 |
| Elasticnet a=0.2 | 0.870956 | 0.932507 | 0.901535 | 0.919056 | 0.907841 | 0.85782 | 0.9283 | 0.910302 |
| Elasticnet a=0.3 | 0.870626 | 0.932783 | 0.900994 | 0.918828 | 0.907922 | 0.85771 | 0.927504 | 0.910161 |
| Elasticnet a=0.4 | 0.870132 | 0.932643 | 0.901668 | 0.919075 | 0.908272 | 0.857066 | 0.927513 | 0.910797 |
| Elasticnet a=0.5 | 0.870151 | 0.932438 | 0.901595 | 0.918302 | 0.908844 | 0.857193 | 0.927698 | 0.910563 |
| Elasticnet a=0.6 | 0.870067 | 0.932452 | 0.901008 | 0.918297 | 0.908618 | 0.85765 | 0.927734 | 0.910335 |
| Elasticnet a=0.7 | 0.869997 | 0.932443 | 0.90079 | 0.918171 | 0.909186 | 0.857207 | 0.928413 | 0.909739 |
| Elasticnet a=0.8 | 0.870286 | 0.932477 | 0.900769 | 0.918244 | 0.909109 | 0.857107 | 0.928651 | 0.909679 |
| Elasticnet a=0.9 | 0.870074 | 0.932549 | 0.90076 | 0.918225 | 0.909329 | 0.857855 | 0.928229 | 0.90971 |
| Lasso (a=1) | 0.869985 | 0.932688 | 0.900646 | 0.91835 | 0.909378 | 0.857492 | 0.928127 | 0.909804 |
| **best model** | **a=0.1 L=0.012** | **a=0.3 L=0.006** | **a=0.4 L=0.065** | **a=0.4 L=0.020** | **a=1 L=0.073** | **a=0.1 L=0.049** | **a=0.8 L=0.008** | **a=0.4 L=0.011** |
| **best result** | **0.871091** | **0.932783** | **0.901668** | **0.919075** | **0.909378** | **0.858805** | **0.928651** | **0.910797** |

BMI, Body mass index; LAP, Lipid accumulation product; BRI, Body roundness index; CVAI, Chinese visceral adiposity index; AVI, Abdominal volume index; BAI, Body adiposity index; TYG, Triglyceride glucose; VAI, Visceral adiposity index; L, lambda.

**Supplementary Table 8.** The result of mean validation AUCs of different models in 10-fold cross-validation in the NCEP-ATPIII criteria, both sex groups.

|  | BMI | LAP | BRI | CVAI | AVI | BAI | TYG | VAI |
| --- | --- | --- | --- | --- | --- | --- | --- | --- |
| Base | 0.780612 | 0.906448 | 0.808274 | 0.858654 | 0.8025 | 0.751693 | 0.90687 | 0.899948 |
| Ridge (a=0) | 0.77956 | 0.898443 | 0.807266 | 0.854653 | 0.801828 | 0.753227 | 0.904963 | 0.89388 |
| Elasticnet a=0.1 | 0.780543 | 0.906334 | 0.808191 | 0.858544 | 0.802778 | 0.754201 | 0.907085 | 0.899895 |
| Elasticnet a=0.2 | 0.780527 | 0.906409 | 0.808329 | 0.858483 | 0.802775 | 0.754119 | 0.907094 | 0.899951 |
| Elasticnet a=0.3 | 0.780574 | 0.906528 | 0.808377 | 0.858435 | 0.802917 | 0.754304 | 0.907214 | 0.899998 |
| Elasticnet a=0.4 | 0.780791 | 0.906721 | 0.808328 | 0.858459 | 0.802969 | 0.754436 | 0.907245 | 0.900054 |
| Elasticnet a=0.5 | 0.780775 | 0.906762 | 0.808331 | 0.858455 | 0.803006 | 0.754689 | 0.907797 | 0.900053 |
| Elasticnet a=0.6 | 0.780755 | 0.90685 | 0.808735 | 0.858453 | 0.803252 | 0.754302 | 0.908042 | 0.900149 |
| Elasticnet a=0.7 | 0.780788 | 0.907029 | 0.808659 | 0.858494 | 0.803362 | 0.754345 | 0.908188 | 0.900337 |
| Elasticnet a=0.8 | 0.780742 | 0.90717 | 0.808617 | 0.858595 | 0.803442 | 0.754531 | 0.908089 | 0.900357 |
| Elasticnet a=0.9 | 0.780726 | 0.907138 | 0.808566 | 0.858666 | 0.803431 | 0.754441 | 0.908061 | 0.900327 |
| Lasso (a=1) | 0.780738 | **0.907173** | 0.808788 | 0.858686 | 0.803566 | 0.754538 | 0.908083 | 0.900614 |
| **best model** | **a=0.4 L=0.003** | **a=1 L=0.003** | **a=1 L=0.008** | **a=1 L=0.002** | **a=1 L=0.017** | **a=0.5 L=0.092** | **a=0.7 L=0.010** | **a=1 L=0.005** |
| **best result** | **0.780791** | **0.907173** | **0.808788** | **0.858686** | **0.803566** | **0.754689** | **0.908188** | **0.900614** |

BMI, Body mass index; LAP, Lipid accumulation product; BRI, Body roundness index; CVAI, Chinese visceral adiposity index; AVI, Abdominal volume index; BAI, Body adiposity index; TYG, Triglyceride glucose; VAI, Visceral adiposity index; L, lambda.

**Supplementary Table 9.** The result of mean validation AUCs of different models in 10-fold cross-validation in the NCEP-ATPIII criteria, male group.

|  | BMI | LAP | BRI | CVAI | AVI | BAI | TYG | VAI |
| --- | --- | --- | --- | --- | --- | --- | --- | --- |
| Base | 0.773182 | 0.908538 | 0.812413 | 0.867934 | 0.818651 | 0.72836 | 0.89727 | 0.898437 |
| Ridge (a=0) | 0.775978 | 0.898082 | 0.813543 | 0.867151 | 0.819793 | 0.733853 | 0.896989 | 0.886484 |
| Elasticnet a=0.1 | 0.776084 | 0.909514 | 0.814484 | 0.868488 | 0.820639 | 0.734194 | 0.897996 | 0.898985 |
| Elasticnet a=0.2 | 0.777826 | 0.909764 | 0.814513 | 0.869269 | 0.821269 | 0.733709 | 0.898213 | 0.899102 |
| Elasticnet a=0.3 | 0.77761 | 0.909888 | 0.814711 | 0.870163 | 0.820823 | 0.735354 | 0.898324 | 0.899317 |
| Elasticnet a=0.4 | 0.778144 | 0.911328 | 0.814433 | 0.869455 | 0.820818 | 0.734301 | 0.898218 | 0.90014 |
| Elasticnet a=0.5 | 0.778721 | 0.912033 | 0.814882 | 0.869776 | 0.820943 | 0.733921 | 0.898417 | 0.901928 |
| Elasticnet a=0.6 | 0.778243 | 0.913015 | 0.814471 | 0.869593 | 0.820881 | 0.733298 | 0.898864 | 0.902551 |
| Elasticnet a=0.7 | 0.778556 | 0.913833 | 0.814383 | 0.869342 | 0.820791 | 0.733271 | 0.898979 | 0.90331 |
| Elasticnet a=0.8 | 0.778155 | 0.914334 | 0.814236 | 0.869221 | 0.820668 | 0.733375 | 0.899518 | 0.903275 |
| Elasticnet a=0.9 | 0.778023 | 0.914414 | 0.814308 | 0.86921 | 0.820704 | 0.733013 | 0.899899 | 0.903855 |
| Lasso (a=1) | 0.778444 | 0.91401 | 0.814435 | 0.869055 | 0.820821 | 0.732892 | 0.900027 | 0.904462 |
| **best model** | **a=0.5 L=0.035** | **a=0.9 L=0.076** | **a=0.5 L=0.016** | **a=0.3 L=0.021** | **a=0.2 L=0.025** | **a=0.3 L=0.118** | **a=1 L=0.010** | **a=1 L=0.065** |
| **best result** | **0.778721** | **0.914414** | **0.814882** | **0.870163** | **0.821269** | **0.735354** | **0.900027** | **0.904462** |

BMI, Body mass index; LAP, Lipid accumulation product; BRI, Body roundness index; CVAI, Chinese visceral adiposity index; AVI, Abdominal volume index; BAI, Body adiposity index; TYG, Triglyceride glucose; VAI, Visceral adiposity index; L, lambda.

**Supplementary Table 10.** The result of mean validation AUCs of different models in 10-fold cross-validation in the NCEP-ATPIII criteria, female group.

|  | BMI | LAP | BRI | CVAI | AVI | BAI | TYG | VAI |
| --- | --- | --- | --- | --- | --- | --- | --- | --- |
| Base | 0.813412 | 0.914478 | 0.829313 | 0.878521 | 0.827238 | 0.802043 | 0.920643 | 0.909758 |
| Ridge (a=0) | 0.812198 | 0.910583 | 0.829621 | 0.872944 | 0.829045 | 0.80339 | 0.918215 | 0.906109 |
| Elasticnet a=0.1 | 0.813385 | 0.914219 | 0.830214 | 0.878451 | 0.828479 | 0.804164 | 0.920956 | 0.909672 |
| Elasticnet a=0.2 | 0.813464 | 0.914297 | 0.830357 | 0.878372 | 0.828649 | 0.803582 | 0.920919 | 0.909656 |
| Elasticnet a=0.3 | 0.813014 | 0.914409 | 0.830394 | 0.878493 | 0.828506 | 0.803688 | 0.920947 | 0.909782 |
| Elasticnet a=0.4 | 0.813089 | 0.914404 | 0.830547 | 0.87852 | 0.828614 | 0.803821 | 0.92098 | 0.909736 |
| Elasticnet a=0.5 | 0.813163 | 0.914352 | 0.830121 | 0.878678 | 0.828214 | 0.803439 | 0.921724 | 0.90998 |
| Elasticnet a=0.6 | 0.813176 | 0.914324 | 0.830087 | 0.878698 | 0.828109 | 0.803407 | 0.921865 | 0.910261 |
| Elasticnet a=0.7 | 0.813237 | 0.914301 | 0.830076 | 0.878823 | 0.828136 | 0.803679 | 0.92193 | 0.91006 |
| Elasticnet a=0.8 | 0.813239 | 0.914398 | 0.830061 | 0.879091 | 0.828195 | 0.803609 | 0.921795 | 0.910177 |
| Elasticnet a=0.9 | 0.813267 | 0.914491 | 0.829955 | 0.878907 | 0.828103 | 0.80372 | 0.921743 | 0.910171 |
| Lasso (a=1) | 0.813119 | 0.914336 | 0.830019 | 0.878862 | 0.828174 | 0.803796 | 0.921764 | 0.910393 |
| **best model** | **a=0.2 L=0.012** | **a=0.9 L=0.005** | **a=0.4 L=0.006** | **a=0.8 L=0.007** | **a=0 L=0.030** | **a=0.1 L=0.011** | **a=0.7 L=0.011** | **a=1 L=0.004** |
| **best result** | **0.813464** | **0.914491** | **0.830547** | **0.879091** | **0.829045** | **0.804164** | **0.92193** | **0.910393** |

BMI, Body mass index; LAP, Lipid accumulation product; BRI, Body roundness index; CVAI, Chinese visceral adiposity index; AVI, Abdominal volume index; BAI, Body adiposity index; TYG, Triglyceride glucose; VAI, Visceral adiposity index; L, lambda

**Supplementary Table 11.** The result of mean validation AUCs of different models in 10-fold cross-validation in the IDF criteria, both sex groups.

|  | BMI | LAP | BRI | CVAI | AVI | BAI | TYG | VAI |
| --- | --- | --- | --- | --- | --- | --- | --- | --- |
| Base | 0.787177 | 0.898377 | 0.824935 | 0.864979 | 0.81312 | 0.752523 | 0.883537 | 0.877663 |
| Ridge (a=0) | 0.786432 | 0.89157 | 0.823773 | 0.860543 | 0.812887 | 0.752333 | 0.881407 | 0.871724 |
| Elasticnet a=0.1 | 0.786722 | 0.898212 | 0.825594 | 0.864815 | 0.813676 | 0.753564 | 0.883514 | 0.877848 |
| Elasticnet a=0.2 | 0.786907 | 0.898756 | 0.825723 | 0.864845 | 0.813653 | 0.754767 | 0.883522 | 0.878494 |
| Elasticnet a=0.3 | 0.787069 | 0.898309 | 0.82589 | 0.864931 | 0.813481 | 0.755383 | 0.88356 | 0.878464 |
| Elasticnet a=0.4 | 0.787125 | 0.898589 | 0.826174 | 0.864937 | 0.813492 | 0.755562 | 0.883562 | 0.877946 |
| Elasticnet a=0.5 | 0.78738 | 0.898667 | 0.826291 | 0.864915 | 0.813801 | 0.755446 | 0.883686 | 0.878214 |
| Elasticnet a=0.6 | 0.787457 | 0.898771 | 0.826271 | 0.864931 | 0.813868 | 0.75536 | 0.883701 | 0.878727 |
| Elasticnet a=0.7 | 0.78769 | 0.898716 | 0.826433 | 0.864928 | 0.814387 | 0.755134 | 0.883853 | 0.879044 |
| Elasticnet a=0.8 | 0.787631 | 0.899187 | 0.826361 | 0.864894 | 0.814575 | 0.754906 | 0.88415 | 0.87904 |
| Elasticnet a=0.9 | 0.787697 | 0.899326 | 0.826126 | 0.864883 | 0.81457 | 0.754833 | 0.884214 | 0.878953 |
| Lasso (a=1) | 0.787815 | 0.899462 | 0.826151 | 0.864954 | 0.814601 | 0.754638 | 0.884339 | 0.879023 |
| **best model** | **a=1 L=0.005** | **a=1 L=0.005** | **a=0.7 L=0.018** | **Base** | **a=1 L=0.016** | **a=0.4 L=0.081** | **a=1 L=0.013** | **a=0.7 L=0.169** |
| **best result** | **0.787815** | **0.899462** | **0.826433** | **0.864979** | **0.814601** | **0.755562** | **0.884339** | **0.879044** |

BMI, Body mass index; LAP, Lipid accumulation product; BRI, Body roundness index; CVAI, Chinese visceral adiposity index; AVI, Abdominal volume index; BAI, Body adiposity index; TYG, Triglyceride glucose; VAI, Visceral adiposity index; L, lambda

**Supplementary Table 12.** The result of mean validation AUCs of different models in 10-fold cross-validation in the IDF criteria, male group.

|  | BMI | LAP | BRI | CVAI | AVI | BAI | TYG | VAI |
| --- | --- | --- | --- | --- | --- | --- | --- | --- |
| Base | 0.795413 | 0.892265 | 0.843256 | 0.887718 | 0.850451 | 0.727138 | 0.860361 | 0.867483 |
| Ridge (a=0) | 0.79553 | 0.884415 | 0.842354 | 0.886792 | 0.848637 | 0.732553 | 0.85981 | 0.854706 |
| Elasticnet a=0.1 | 0.804581 | 0.892674 | 0.848104 | 0.888234 | 0.853324 | 0.739526 | 0.861331 | 0.869574 |
| Elasticnet a=0.2 | 0.804621 | 0.893876 | 0.848707 | 0.888251 | 0.853755 | 0.740039 | 0.861705 | 0.868764 |
| Elasticnet a=0.3 | 0.805167 | 0.894451 | 0.848696 | 0.888241 | 0.854384 | 0.739289 | 0.861599 | 0.870385 |
| Elasticnet a=0.4 | 0.804167 | 0.895063 | 0.848325 | 0.888114 | 0.854291 | 0.739184 | 0.861858 | 0.8706 |
| Elasticnet a=0.5 | 0.804868 | 0.896407 | 0.848838 | 0.888 | 0.854378 | 0.738783 | 0.861802 | 0.870686 |
| Elasticnet a=0.6 | 0.804422 | 0.896985 | 0.848849 | 0.887878 | 0.854583 | 0.738719 | 0.862483 | 0.872372 |
| Elasticnet a=0.7 | 0.80458 | 0.897536 | 0.848722 | 0.887774 | 0.8548 | 0.739119 | 0.862453 | 0.871639 |
| Elasticnet a=0.8 | 0.804371 | 0.899708 | 0.848614 | 0.887774 | 0.855172 | 0.739524 | 0.862676 | 0.871911 |
| Elasticnet a=0.9 | 0.804484 | 0.900464 | 0.848603 | 0.887665 | 0.854807 | 0.739497 | 0.863029 | 0.87214 |
| Lasso (a=1) | 0.804476 | 0.90068 | 0.84887 | 0.887686 | 0.854289 | 0.739391 | 0.863094 | 0.87299 |
| **best model** | **a=0.3 L=0.22** | **a=1 L=0.055** | **a=1 L=0.041** | **a=0.2 L=0.004** | **a=0.8 L=0.037** | **a=0.2 L=0.185** | **a=1 L=0.006** | **a=1 L=0.040** |
| **best result** | **0.805167** | **0.90068** | **0.84887** | **0.888251** | **0.855172** | **0.740039** | **0.863094** | **0.87299** |

BMI, Body mass index; LAP, Lipid accumulation product; BRI, Body roundness index; CVAI, Chinese visceral adiposity index; AVI, Abdominal volume index; BAI, Body adiposity index; TYG, Triglyceride glucose; VAI, Visceral adiposity index; L, lambda

**Supplementary Table 13.** The result of mean validation AUCs of different models in 10-fold cross-validation in the IDF criteria, female group.

|  | BMI | LAP | BRI | CVAI | AVI | BAI | TYG | VAI |
| --- | --- | --- | --- | --- | --- | --- | --- | --- |
| Base | 0.815583 | 0.911427 | 0.839996 | 0.883096 | 0.836704 | 0.808117 | 0.905012 | 0.893843 |
| Ridge (a=0) | 0.815592 | 0.908541 | 0.839007 | 0.876491 | 0.838279 | 0.80919 | 0.902228 | 0.890835 |
| Elasticnet a=0.1 | 0.816091 | 0.912579 | 0.839849 | 0.883469 | 0.838534 | 0.809008 | 0.905383 | 0.894859 |
| Elasticnet a=0.2 | 0.816013 | 0.91275 | 0.83998 | 0.883384 | 0.838348 | 0.808646 | 0.905382 | 0.895133 |
| Elasticnet a=0.3 | 0.815844 | 0.912848 | 0.840096 | 0.883262 | 0.838389 | 0.809141 | 0.905467 | 0.895656 |
| Elasticnet a=0.4 | 0.816064 | 0.913046 | 0.839814 | 0.883428 | 0.838235 | 0.809139 | 0.905445 | 0.89565 |
| Elasticnet a=0.5 | 0.816183 | 0.913072 | 0.840139 | 0.88341 | 0.8383 | 0.809383 | 0.905333 | 0.895917 |
| Elasticnet a=0.6 | 0.81604 | 0.912856 | 0.840424 | 0.883327 | 0.837819 | 0.809722 | 0.905756 | 0.896034 |
| Elasticnet a=0.7 | 0.816042 | 0.912847 | 0.840511 | 0.883407 | 0.83771 | 0.809534 | 0.905743 | 0.89627 |
| Elasticnet a=0.8 | 0.81638 | 0.913096 | 0.840448 | 0.883383 | 0.837603 | 0.809479 | 0.906001 | 0.896308 |
| Elasticnet a=0.9 | 0.816279 | 0.913112 | 0.840271 | 0.883355 | 0.83773 | 0.809625 | 0.906189 | 0.896284 |
| Lasso (a=1) | 0.81623 | 0.913187 | 0.840005 | 0.883496 | 0.838005 | 0.809582 | 0.906535 | 0.896478 |
| **best model** | **a=0.8 L=0.006** | **a=1** **L=0.006** | **a=0.7** **L=0.029** | **a=1** **L=0.002** | **a=0.1** **L=0.05** | **a=0.6** **L=0.015** | **a=1** **L=0.014** | **a=1** **L=0.004** |
| **best result** | **0.81638** | **0.913187** | **0.840511** | **0.883496** | **0.838534** | **0.809722** | **0.906535** | **0.896478** |

BMI, Body mass index; LAP, Lipid accumulation product; BRI, Body roundness index; CVAI, Chinese visceral adiposity index; AVI, Abdominal volume index; BAI, Body adiposity index; TYG, Triglyceride glucose; VAI, Visceral adiposity index; L, lambda

**Supplementary Table 14.** The cut-off, sensitivities, specificities and Youden’s index of each variable for the screening of metabolic syndrome in the NCEP-ATPIII criteria.

| Group | Variable | Optimal Cut-Offs | Youden Index | Sensitivity (%) | Specificity (%) |
| --- | --- | --- | --- | --- | --- |
| All | BMI | 24.1 | 0.364 | 76.3 | 60.1 |
|  | LAP | 41.02 | 0.632 | 81.8 | 81.4 |
|  | BRI | 3.98 | 0.418 | 75.2 | 66.6 |
|  | CVAI | 113.71 | 0.518 | 81.1 | 70.7 |
|  | AVI | 15.97 | 0.385 | 66.8 | 71.7 |
|  | BAI | 28.35 | 0.228 | 69.9 | 52.9 |
|  | TYG | 8.78 | 0.613 | 79.3 | 82 |
|  | VAI | 1.77 | 0.593 | 81.3 | 78 |
| Male | BMI | 24.01 | 0.381 | 84.3 | 53.8 |
|  | LAP | 41.22 | 0.677 | 88.3 | 79.4 |
|  | BRI | 3.97 | 0.468 | 83.2 | 63.6 |
|  | CVAI | 116.80 | 0.541 | 91.9 | 62.2 |
|  | AVI | 16.23 | 0.491 | 91.9 | 57.2 |
|  | BAI | 27.46 | 0.278 | 55.8 | 72.0 |
|  | TYG | 8.85 | 0.616 | 80.7 | 80.9 |
|  | VAI | 1.77 | 0.617 | 78.2 | 83.5 |
| Female | BMI | 24.11 | 0.350 | 70.9 | 64.1 |
|  | LAP | 40.49 | 0.606 | 77.9 | 82.7 |
|  | BRI | 3.42 | 0.416 | 88.8 | 52.8 |
|  | CVAI | 104.67 | 0.567 | 85.7 | 71.0 |
|  | AVI | 12.93 | 0.434 | 95.0 | 48.4 |
|  | BAI | 32.14 | 0.280 | 49.6 | 78.4 |
|  | TYG | 8.78 | 0.624 | 76.7 | 85.7 |
|  | VAI | 1.88 | 0.589 | 81.4 | 77.5 |

BMI, Body mass index; LAP, Lipid accumulation product; BRI, Body roundness index; CVAI, Chinese visceral adiposity index; AVI, Abdominal volume index; BAI, Body adiposity index; TYG, Triglyceride glucose; VAI, Visceral adiposity index.

**Supplementary Table 15.** The cut-off, sensitivities, specificities and Youden’s index of each variable for the screening of metabolic syndrome in the IDF criteria

| Group | Variable | Optimal Cut-Offs | Youden Index | Sensitivity (%) | Specificity (%) |
| --- | --- | --- | --- | --- | --- |
| All | BMI | 24.1 | 0.401 | 79.5 | 60.6 |
|  | LAP | 40.49 | 0.615 | 82.3 | 79.2 |
|  | BRI | 3.98 | 0.476 | 80.0 | 67.6 |
|  | CVAI | 113.71 | 0.552 | 84.4 | 70.8 |
|  | AVI | 15.97 | 0.438 | 71.2 | 72.6 |
|  | BAI | 28.35 | 0.26 | 72.6 | 53.4 |
|  | TYG | 8.77 | 0.578 | 78.6 | 79.2 |
|  | VAI | 1.77 | 0.56 | 80.0 | 76.0 |
| Male | BMI | 24.01 | 0.451 | 90.1 | 55.0 |
|  | LAP | 41.22 | 0.648 | 87.9 | 76.9 |
|  | BRI | 3.97 | 0.558 | 90.7 | 65.1 |
|  | CVAI | 127.15 | 0.611 | 86.3 | 74.8 |
|  | AVI | 16.23 | 0.589 | 1.0 | 58.9 |
|  | BAI | 27.46 | 0.334 | 60.4 | 73.0 |
|  | TYG | 8.85 | 0.572 | 79.1 | 78.1 |
|  | VAI | 1.77 | 0.570 | 76.4 | 80.6 |
| Female | BMI | 24.11 | 0.368 | 72.6 | 64.2 |
|  | LAP | 39.32 | 0.601 | 81.5 | 78.6 |
|  | BRI | 3.45 | 0.457 | 91.9 | 53.8 |
|  | CVAI | 104.67 | 0.560 | 85.9 | 70.1 |
|  | AVI | 12.85 | 0.486 | 1.0 | 48.6 |
|  | BAI | 32.14 | 0.304 | 51.6 | 78.8 |
|  | TYG | 8.78 | 0.594 | 75.4 | 84.0 |
|  | VAI | 1.88 | 0.536 | 80.2 | 76.1 |

BMI, Body mass index; LAP, Lipid accumulation product; BRI, Body roundness index; CVAI, Chinese visceral adiposity index; AVI, Abdominal volume index; BAI, Body adiposity index; TYG, Triglyceride glucose; VAI, Visceral adiposity index.

**Supplementary Table 16.** Pairwise comparisons of the AUCs of eight obesity- and lipid-related indices in predicting metabolic syndrome by the Delong test with the China (2020) criteria.

| Variable | BMI | LAP | BRI | CVAI | AVI | BAI | TYG |
| --- | --- | --- | --- | --- | --- | --- | --- |
| LAP | 9.67^**^ |  |  |  |  |  |  |
| BRI | 4.859^**^ | 6.7^**^ |  |  |  |  |  |
| CVAI | 9.162^**^ | 3.126^*^ | 6.243^**^ |  |  |  |  |
| AVI | 6.511^**^ | 5.464^**^ | 2.519^*^ | 4.859^**^ |  |  |  |
| BAI | 9.502^**^ | 15.333^**^ | 14.347^**^ | 14.326^**^ | 11.7^**^ |  |  |
| TYG | 6.978^**^ | 2.826^*^ | 4.202^**^ | 1.01 | 3.132^*^ | 13.214^**^ |  |
| VAI | 5.334^**^ | 6.489^**^ | 2.578^*^ | 0.81 | 1.491 | 11.889^**^ | 3.379^**^ |

**P< 0.001, *P< 0.05

**Supplementary Table 17.** Pairwise comparisons of the AUCs of eight obesity- and lipid-related indices in predicting metabolic syndrome by the Delong test with the NCEP-ATPIII criteria.

|  | BMI | LAP | BRI | CVAI | AVI | BAI | TYG |
| --- | --- | --- | --- | --- | --- | --- | --- |
| LAP | 12.077^**^ |  |  |  |  |  |  |
| BRI | 4.993^**^ | 9.142^**^ |  |  |  |  |  |
| CVAI | 9.681^**^ | 5.452^**^ | 6.415^**^ |  |  |  |  |
| AVI | 3.13^*^ | 10.61^**^ | 2.627^*^ | 9.021^**^ |  |  |  |
| BAI | 5.122^**^ | 14.213^**^ | 9.496^**^ | 11.096^**^ | 6.071^**^ |  |  |
| TYG | 9.488^**^ | 1.495 | 6.56^**^ | 3.468^**^ | 7.663^**^ | 12.619^**^ |  |
| VAI | 8.53^**^ | 3.388^**^ | 5.622^**^ | 2.409^*^ | 6.616^**^ | 12.177^**^ | 1.863 |

**P< 0.001, *P< 0.05

**Supplementary Table 18.** Pairwise comparison of the AUCs of eight obesity- and lipid-related indices in predicting metabolic syndrome by the Delong test with the IDF criteria.

| Variable | BMI | LAP | BRI | CVAI | AVI | BAI | TYG |
| --- | --- | --- | --- | --- | --- | --- | --- |
| LAP | 10.156^**^ |  |  |  |  |  |  |
| BRI | 5.667^**^ | 6.461^**^ |  |  |  |  |  |
| CVAI | 8.784^**^ | 3.649^**^ | 4.595^**^ |  |  |  |  |
| AVI | 3.341^**^ | 8.363^**^ | 3.317^**^ | 7.776^**^ |  |  |  |
| BAI | 4.958^**^ | 12.428^**^ | 9.889^**^ | 10.306^**^ | 6.07^**^ |  |  |
| TYG | 6.604^**^ | 4.152^**^ | 3.145^*^ | 0.629 | 4.612^**^ | 9.858^**^ |  |
| VAI | 5.997^**^ | 5.588^**^ | 2.553^*^ | 0.0143 | 3.945^**^ | 9.688^**^ | 1.213 |

**P< 0.001, *P< 0.05

## 2.Supplementary Figures


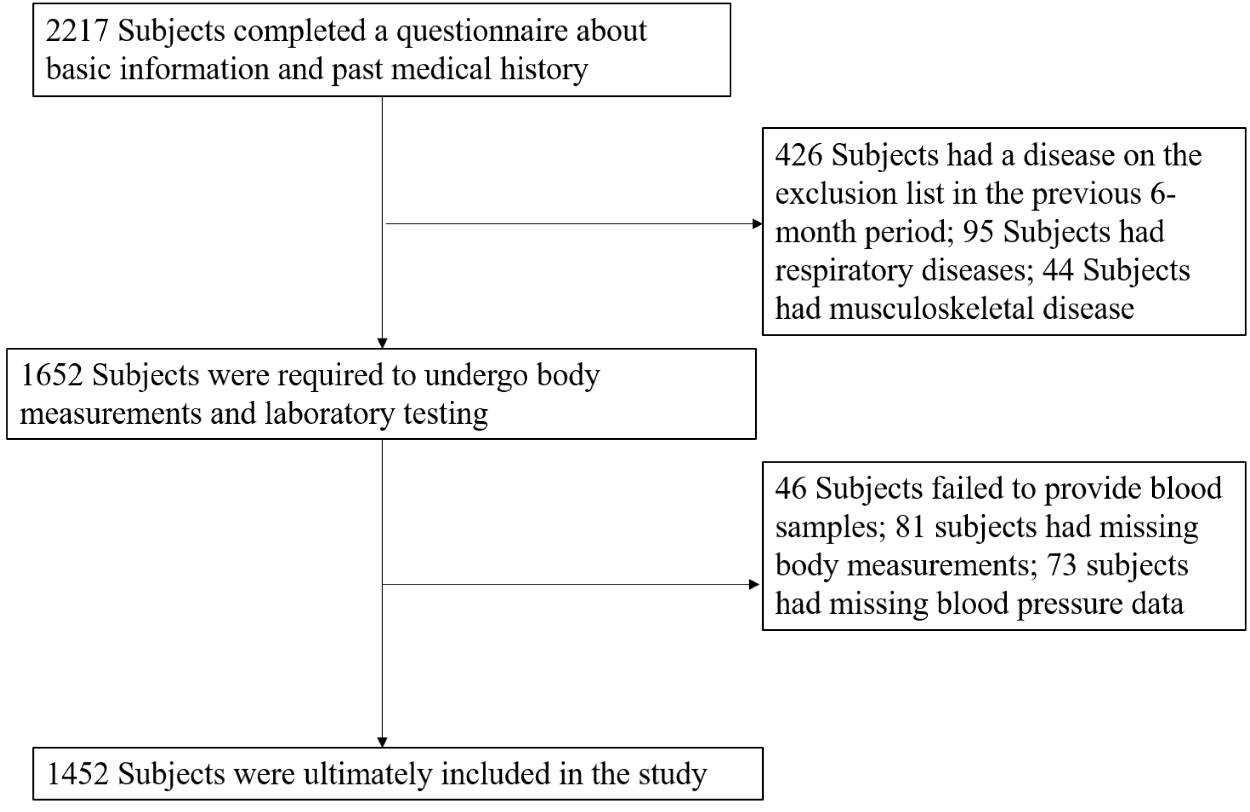


**Supplementary Figure 1**. The flow diagram shows the screening and enrollment of the participants.
